# Efficacy and safety of rt-PA intravenous thrombolysis in patients with wake-up stroke: A meta-analysis

**DOI:** 10.1097/MD.0000000000028914

**Published:** 2022-02-18

**Authors:** Hongfa Liu, Weihua Hu, Fang Zhang, Wei Gu, Jiankun Hong, Jianping Chen, Ying Huang, Huoying Pan

**Affiliations:** a The First Clinical College of Gannan Medical University, Ganzhou, Jiangxi, China; b Department of Geriatrics, The Third People's Hospital of Ganzhou, Ganzhou, Jiangxi, China; c Department of Neurology, Ganxian People's Hospital, Ganzhou, Jiangxi, China; d Department of General Practice, Ganzhou People's Hospital, Ganzhou, Jiangxi, China; e Key Laboratory of Prevention and Treatment of Cardiovascular and Cerebrovascular Diseases, Ministry of Education, Gannan Medical University, Ganzhou, Jiangxi, China; f Department of Neurology, The First Affiliated Hospital of Gannan Medical University, Ganzhou, Jiangxi, China; g Gannan Branch Center of National Geriatric Disease Clinical Medical Research Center, Gannan Medical University, Ganzhou, Jiangxi, China.

**Keywords:** efficacy, meta-analysis, safety, thrombolysis, wake-up stroke

## Abstract

**Background:**

: Recombinant tissue plasminogen activator (rt-PA) is one of the most effective therapies for patients with acute ischemic stroke. However, wake-up stroke (WUS) is typically excluded from intravenous thrombolytic therapy because of the unclear time of symptom onset. Therefore, we aimed to assess the efficacy and safety of rt-PA intravenous thrombolysis in patients with WUS by meta-analysis.

**Methods:**

: We completed a systematic literature search of PubMed, Embase, the Cochrane Library, and SinoMed and included relevant studies of WUS patients covering rt-PA thrombolysis and nonthrombolysis (published from January 1, 2000, to February 28, 2021, with no language restrictions). The primary outcomes included safety outcomes and functional outcomes. Safety outcomes were measured according to the incidence of symptomatic intracranial hemorrhage and mortality within 90 days. The efficacy outcomes were measured based on 90-day modified Rankin Scale scores. We assessed pooled data using either a random-effects model (when *P* < .10, *I* ^2^ > 50%) or a fixed-effects model (when *P* > .10, *I* ^2^ < 50%).

**Results:**

: A total of 913 patients from 9 studies were included in the meta-analysis. All patients had ischemic stroke confirmed by computed tomography or magnetic resonance imaging. The incidence of modified Rankin Scale 0 to 2 was significantly higher in the rt-PA thrombolysis group compared with the nonthrombolysis group. And rt-PA thrombolytic WUS patients did not differ significantly from nonthrombolytic WUS patients in terms of 90-day mortality. However, the incidence of Symptomatic intracranial hemorrhage was also significantly higher in the rt-PA thrombolysis group than that in the nonthrombolysis group.

**Conclusions:**

: Patients with WUS who received rt-PA thrombolysis had a significant positive effect within 90 days. In addition, although there was no significant increase in mortality, we need to be aware of the risk of intracranial hemorrhage transformation associated with rt-PA thrombolysis despite no obvious increase in mortality. The safety of rt-PA intravenous thrombolysis should be closely monitored in patients with WUS.

## 1 Introduction

Acute ischemic stroke is the second leading cause of death and the third leading cause of disability in the world.^[1]^ In China, stroke is a major cause of mortality as well as long-term physical and cognitive impairment. Approximately one-fifth of ischemic strokes occur during sleep and are not detected until patients wake up with neurological deficits.^[^2^,^^3^^]^ Stroke Screening and Prevention Program could improve the effective control rate of primary diseases in high-risk groups.^[4]^ However, Recombinant tissue plasminogen activator (rt-PA) is currently the unique approved treatment for acute ischemic stroke and is restricted to use within 4.5 hours of ischemic stroke onset.^[5]^ We typically define the last seen normal time as the time of stroke onset. Therefore, WUS has historically been considered a contraindication for rt-PA intravenous thrombolysis.^[^6^,^^7^^]^

However, a growing number of clinical and imaging observations have demonstrated that a large number of WUS events occur in the early morning and close to the wake-up time.^[^8^,^^9^^]^ WUS has similar imaging and clinical characteristics to strokes of known onset times. Moreover, in recent years, several studies have found rt-PA intravenous thrombolysis to be safe and effective in imaging-mismatched patients with WUS.^[^10^,^^11^^]^ Although the benefit of rt-PA intravenous thrombolysis in patients with WUS remains controversial, we conducted a meta-analysis of all relevant studies to evaluate the efficacy and safety of rt-PA intravenous thrombolysis in patients with WUS.

## 2 Methods

### 2.1 Search strategy

We clearly defined the themes of our systematic review in terms of population, interventions, comparators, outcomes and study design (PICOS). patients with WUS who were the study population were divided into 2 groups: 1 group received rt-PA intravenous thrombolysis and the control group received conventional therapy outcomes included mortality, occurrence of symptomatic intracerebral hemorrhage (SICH) and modified Rankin Scale (mRS) scores within 90 days. PubMed, Embase, The Cochrane Library and SinoMed were searched from January 1, 2000, to June 30, 2021. A combination of the following terms was used: “wake up stroke,” “wake-up stroke,” “stroke on awakening,” “wake up,” “recombinant tissue plasminogen activator,” “alteplase,” “rtPA,” “rt-PA,” and “intravenous thrombolysis,” “thrombolysis,” “reperfusion” and “thrombolytic therapy.” For example, the search strategy of PubMed was (intravenous thrombolysis OR thrombolysis OR reperfusion OR thrombolytic therapy) AND (recombinant tissue plasminogen activator OR alteplase OR rtPA OR rt-PA) AND (wake up stroke OR wake-up stroke OR stroke on awakening OR wake up). We also conducted a manual search. We then screened studies based on title, abstract, and full-text reading. The search strategy was developed without any language restrictions. Given that the study was based on published articles, ethical approval and patient consent were not required.

### 2.2 Inclusion and exclusion criteria

According to the PICOS principle, all studies published in peer-reviewed journals were listed according to the following criteria:

1. WUS patients with no restrictions on gender, age, race, or nationality,
2. study population included WUS patients who received rt-PA intravenous thrombolysis (total of 0.9 mg/kg rt-PA),
3. matched controls were WUS patients who were not treated with rt-PA intravenous thrombolysis or received standard treatment,
4. the efficacy of rt-PA intravenous thrombolysis was assessed with a 90-day mRS score. Safety was measured by mortality and SICH within 90 days after rt-PA intravenous thrombolysis, and
5. studies had intact data and were published.

The exclusion criteria were as follows:

1. conference proceedings, correspondence, case reports, reviews, editorials or preclinical studies,
2. studies lacking critical key information, such as no control group, loss of therapeutic details and basic characteristics of patients,
3. studies with repeated report analysis, and studies lacking important data.
4. Studies with a score of less than 6 assessed with the Newcastle-Ottawa Scale (NOS).^[12]^

### 2.3 Quality assessment

We used the NOS to evaluate the non-randomized studies. The NOS included 3 items: case selection, comparability, and exposure assessment. Each project was evaluated one by one, and finally the studied quality was classified into 3 levels based on the total number of stars: low quality (0–3 stars), medium quality (4–6 stars), and high quality (7–9 stars). Two researchers (H. F. Liu and Y. Huang) performed quality assessments independently, and resolved differences by consensus.

### 2.4 Data extraction

Data were extracted by 2 authors independently. Any divergence was resolved by discussion between the 2 authors and determined eventually by the senior author. Data were collected on authors, publication year, trial design, study period, intervention and comparisons, participant features (number, age, sex, disease history, Toast classification, neuroimaging methods, and NIHSS scores), comorbidities (transient ischemic attack, atrial fibrillation, coronary heart disease, hypertension, and diabetes mellitus), NIHSS scores, admission time and clinical endpoints, including mRS, mortality and the rate of SICH, were collected.

### 2.5 Outcome measures

Safety outcomes measures included the incidence of symptomatic intracranial hemorrhage (SICH) and mortality within 90 days. Efficacy outcomes were measured based on 90-day mRS scores.

### 2.6 Statistical analysis

All results were presented as odds ratios (ORs) and 95% confidence intervals (CIs). The heterogeneity of the studies was evaluated by the chi-squared test and qualified by *I* ^2^ statistics. When *I* ^2^ < 50% and *P* > .10, a fixed effects model was used to analyze the results, which demonstrated low heterogeneity among studies. Otherwise, a random effects model was applied in the analysis. A two-sided *P* values of .05 were considered statistically significant. Publication bias was assessed by funnel plots. Sensitivity analyses were conducted to elucidate the effect of each individual study on the overall estimate by removing each study in turn. All statistical analyses were performed using Review Manager (RevMan, version 5.3.5).

## 3 Results

### 3.1 Study selection

**Figure 1:**
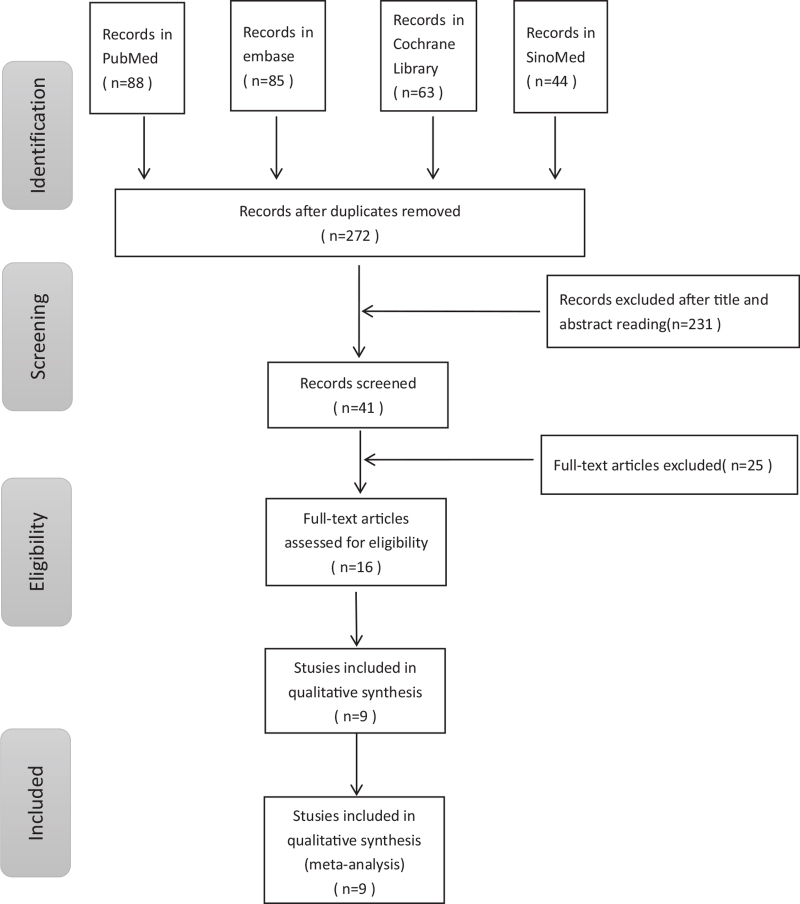
Flowchart of the study selection.

The flowchart of the systematic literature search is presented in Figure 1. We identified potentially relevant studies, including 88 from PubMed, 85 from Embase, 63 from Cochrane, and 44 from SinoMed. A total of 271 studies were excluded after reading the title, abstract and full-text using the formulated inclusion and exclusion criteria. Finally, a total of 9 studies^[^11^,^^13^^–^20^]^ with 913 patients, including 376 patients (376/913, 41.18%) treated with rt-PA thrombolysis, were included in the meta-analysis. Nine studies were all retrospective. The baseline characteristics of the WUS patients included in these studies are summarized in Table 1. There were no significant differences between the WUS thrombolysis group and the WUS nonthrombolysis group were noted in terms of age, sex, previous history or risk factors of stroke patients.

**Table 1:** The baseline characteristics of the included studies.

Study	Anaissie 2016^[13]^	Bal 2014^[14]^	Barreto 2009^[15]^	Breuer 2010^[11]^	Furlanis 2019^[16]^	Liu 2018^[20]^	Li 2016^[19]^	Manawadu 2013^[17]^	Roveri 2013^[18]^
Design	Retrospective	Retrospective	Retrospective	Retrospective	Retrospective	Retrospective	Retrospective	Retrospective	Retrospective
Period	2008.07–2014.05	2003.01–2010.03	2003.03–2008.01	2006.10–2008.05	2013.11–2018.12	2016.05–2018.06	2015.06–2016.06	2009.01–2010.12	2005.02–2010.07
No.of patients	46/154	29/41	46/34	10/35	74/75	30/35	64/58	68/54	9/51
Age(y)	69 (42–98)/63 (22–93)^†^	68 (23)/74 (21)^‡^	62 ± 14/64 ± 13^∗^	73 (57–92)/66 (39-87)^†^	73 ± 13/79 ± 10^∗^	66.0 ± 9.3/65.8 ± 10.8^∗^	62.9 ± 2.1/65.9 ± 11.3^∗^	Not reported	59.22 (13.13)/70.43 (10.28)^‡^
Gender (male%)	22 (47.9)/85 (55.2)	12 (41)/17 (41)	11 (39)/15 (44)	5 (50)/23 (66)	30 (41)/39 (52)	18 (60.0/20 (57.1)	38 (59.4)/33 (56.9)	23 (33.8)/28 (51.9)	33 (55)/48 (55.8)
Admission NIHSS score	9.5 (1-27)/5 (0-33)^†^	14 (11)/13 (11)^‡^	16 (3–24)/10.5 (2-26)^†^	10.5 (1–22)/6 (1-21)^†^	6 (4–13)/7 (3-16)^†^	Not reported	8.7 ± 5.2/9.2 ± 4.9^∗^	11 (8-17)/9.5 (5–16)^†^	9 (4–19)/7 (4–18)^†^
Hypertension (n%)	36 (78.3)/119 (77.3)	18 (62.1)/27 (65.9)	30 (65)/22 (65)	9 (90)/29 (83)	53 (72)/66 (88)	19 (63.3)/23 (65.7)	25 (39.1)/23 (39.7)	45 (66.2)/32 (59.3)	36 (60)/55 (64)
Diabetes mellitus (n%)	20 (43.5)/45 (29.2)	3 (10.3)/7 (17.1)	10 (21)/11 (33)	4 (40)/13 (37)	15 (20)/26 (35)	8 (26.7)/11 (31.4)	20 (31.3)/19 (32.8)	14 (20.6)/13 (24.1)	14 (23.3)/10 (11.6)
Cardiovascular disease (n%)	Not reported	Not reported	8 (17)/5 (15)	3 (30)/6 (17)	15 (20)/22 (29)	10 (33.3)/13 (37.1)	23 (35.9)/21 (36.2)	Not reported	24 (40)/45 (52.3)
Smoking (n%)	Not reported	13 (44.8)/19 (46.3)	Not reported	Not reported	15 (20)/21 (25)	10 (33.3)/14 (40.0)	29 (45.3)/25 (43.1)	Not reported	12 (20.7)/21 (25.9)
Arterial fibrillation (n%)	6 (13)/21 (13.6)	10 (34.5)/18 (43.9)	Not reported	3 (30)/6 (17)	18 (24)/40 (53)	3 (10.0)/5 (14.3)	7 (10.9)/7 (12.1)	21 (30.9)/9 (16.7)	Not reported
Previous stroke/transient ischemic attack (n%)	Not reported	Not reported	Not reported	5 (50)/9 (26)	Not reported	3 (10.0)/5 (14.3)	Not reported	Not reported	14 (23.3)/21 (24.4)
Dyslipidemia (n,%)	19 (41.3)/60 (39)	9 (31.0)/6 (14.6)	12 (27)/9 (27)	8 (80)/27 (77)	47 (64)/47 (63)	18 (60.0)/20 (57.1)	18 (28.1)/15 (25.9)	22 (32.4)/25 (46.3)	19 (31.7)/30 (34.9)
Imaging criteria	Early ischemic changes of <1/3 Middle cerebral artery (MCA) territory	Alberta Stroke Program Early Computed Tomography Scores (ASPECTS) > 7 on Computed tomography	Early ischemic changes of <1/3 MCA territory	Perfusion weighted imaging (PWI)/Diffusion weighted imagin (DWI) mismatch:DWI volume<1/3 MCA territory	ASPECTS > 6 on Computed tomography and/or ischemic penumbra > 50% of hypoperfused tissue on Computed tomography perfusion	DWI/Fluid attenuated inversion recovery (FLAIR) mismatch	DWI/FLAIR mismatch	Early ischemic changes of <1/3 MCA territory	Early ischemic changes of <1/3 MCA territory
Admission time (h)	4.5	Not reported	3	6	4	4.5	4.5	4.5	3

∗ mean ± SD.

† median (minimum- maximum).

‡ mean (interquartile).

### 3.2 Estimation of study quality

After screening of study, 9 studies were included in the meta-analysis and we did not include the randomized research. Quality was evaluated according to the NOS evaluation scale, with 6 high-quality studies (above 7 stars) and 3 of medium quality (4–6 stars), and overall studies were of high quality. The results of the quality evaluation are simultaneously presented in Table 2.

**Table 2:** The study quality evaluation scores.

Study ID	Study selection (4 stars)	Comparability between groups (2 stars)	Outcome (3 stars)	Newcastle-Ottawa scale score (9 stars)
Anaissie 2016^[13]^	^★★★^	^★★^	^★★^	^★★★★★★★^
Bal 2014^[14]^	^★★★^	^★★^	^★★★^	^★★★★★★★★^
Barreto 2009^[15]^	^★★^	^★^	^★★★^	^★★★★★★^
Breuer 2010^[11]^	^★★★^	^★★^	^★★★^	^★★★★★★★★^
Furlanis 2019^[16]^	^★★★★^	^★★^	^★★★^	^★★★★★★★★★^
Liu 2018^[20]^	^★★★^	^★★^	^★^	^★★★★★★^
Li 2016^[19]^	^★★★^	^★★^	^★★★^	^★★★★★★★★^
Manawadu 2013^[17]^	^★★★^	^★★^	^★★★^	^★★★★★★★★^
Roveri 2013^[18]^	^★★★^	^★★^	^★★^	^★★★★★★★^

### 3.3 Outcome assessment

**Figure 2:**
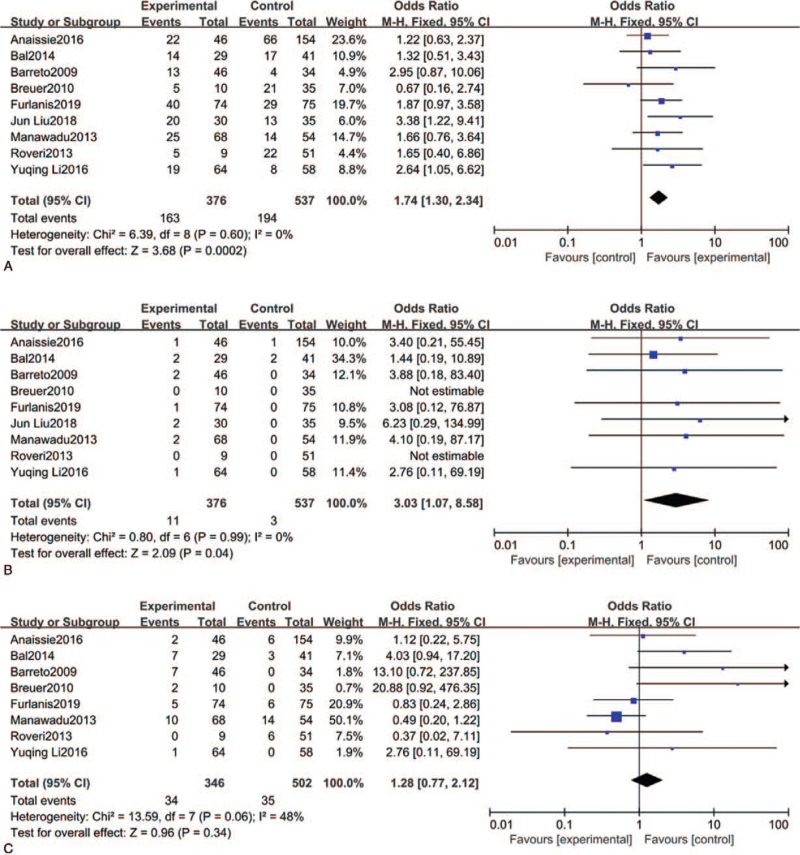
(A). Forest plot of the rate of mRS (0-2) within 90 days between experimental group and control group. (B). Forest plot of the rate of SICH within 90 days between experimental group and control group. (C). Forest plot of the mortality within 90 days between experimental group and control group.

All results are shown in Figure 2. WUS patients who received rt-PA intravenous thrombolysis presented a higher rate of 90-day mRS 0–2 than nonthrombolysis WUS patients (43.35% vs 36.13%). The overall analysis of 90-day mRS 0–2 outcome showed a pooled OR of 1.74 (95% CI 1.30–2.34, *P* = .0002). For safety outcomes, 8 studies including 848 patients reported 90-day mortality. We did not identify a significant difference in 90-day mortality (9.83% vs 6.97%) with a pooled OR of 1.53 (95% CI 0.63–3.76, *P* = .35). However, the incidence of SICH was significantly higher in the rt-PA intravenous thrombolysis group compared with the group that did not receive rt-PA intravenous thrombolysis (2.93% vs 0.56%) with a pooled OR of 3.03 (95% CI 1.07–8.58, *P* = .04).

### 3.4 Heterogeneity, sensitivity analysis, and publication bias assessment

**Figure 3:**
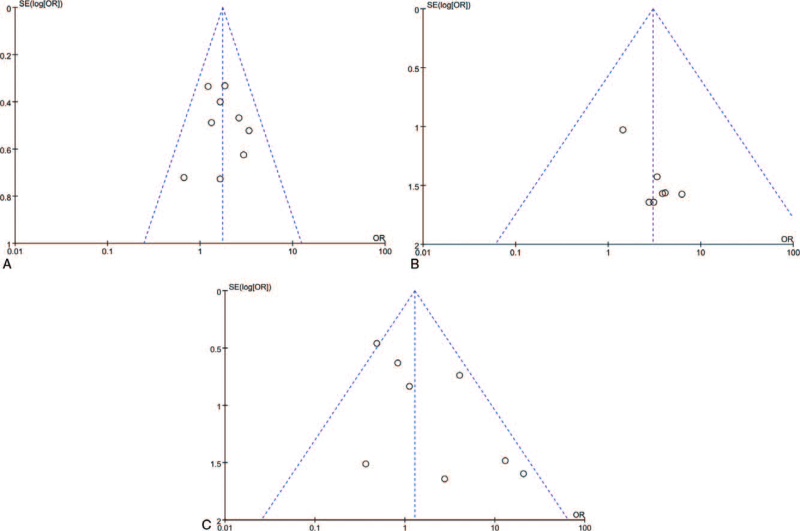
(A). Funnel plot of the rate of mRS(0-2) within 90 days between experimental group and control group. (B). Funnel plot of the rate of SICH within 90 days between experimental group and control group. (C). Funnel plot of the mortality within 90 days between experimental group and control group.

There was no obvious heterogeneity among the results of 90-day mRS 0-2, 90-day mRS 0-1 and the incidence of SICH. However, there was relatively high heterogeneity was noted within 90-day mortality (*I* ^2^ = 48%; *P* = .06). Therefore, we used the leave-one-out method to perform sensibility analysis. We found no significant changes in primary outcomes of 90-day mortality, the rate of SICH, the proportion of mRS 0–2 after extracting each study individually. In these included studies, we did not identify significant publication bias because the funnel plots were symmetrically distributed (Fig. 3).

## 4 Discussion

Based on previous experience, patients with unknown time of stroke onset, including WUS, were ineligible for thrombolysis treatment according to routine clinical practice guidelines.^[21]^ Nevertheless, in recent years, traditional concepts have been gradually shattered based on the generation and development of new diagnostic neuroimaging techniques.^[22]^ The time window for evaluating the presence of ischemic penumbra in tissue has gradually taken the place of the traditional time window. This viewpoint has implications for intravenous thrombolysis for ischemic stroke of unknown duration, including WUS patients. As demonstrated in the EXTEND trial,^[23]^patients with ischemic stroke who received intravenous thrombolysis between 4.5 and 9.0 hours after stroke onset or when they awoke from stroke onset symptoms had improved functional outcomes at 90 days. Similar findings were confirmed in the WAKE-UP trial,^[24]^ , in which patients who received intravenous thrombolysis had favorable functional outcomes at 90 days compared with placebo. These patients had an unknown time of stroke onset, and there was a magnetic resonance imaging (MRI) mismatch, and MRI findings of an ischemic focus on diffusion-weighted imaging with no significant visible signal changes in the corresponding region on fluid attenuation inversion recovery imaging. In addition, the 2019 American Stroke Association guideline recommended that rt-PA intravenous thrombolysis could benefit WUS patients when the onset of deficit symptoms is close to awakening or when there is a brain imaging mismatch and given IIb recommendations.^[25]^ Our meta-analysis also demonstrated that WUS patients treated with rt-PA intravenous thrombolysis had a higher rate of good 90-day outcomes (mRS 0–2) without increased mortality compared with nonthrombolysis WUS patients. Thus, we can consider that rt-PA intravenous thrombolysis in WUS patients is moderately effective. However, it is still regretful that the WAKE-UP and EXTEND trials were not included in our meta-analysis because not all patients in these 2 trials were WUS patients, which did not meet our inclusion criteria.

To our knowledge, similar articles have been published in the last 2 years. The difference is that their research subjects were stroke patients with unknown onset times rather than WUS.^[^26^,^^27^^]^ In addition, rt-PA intravenous thrombolysis increased the incidence of SICH in WUS patients in our final outcome assessment, which was inconsistent with previous similar research findings. However, we do not believe that rt-PA intravenous thrombolysis treatment is unreliable for WUS patients. There are several possible reasons for the different results. On the 1 hand, standard imaging inclusion criteria were not employed among the nine included studies, and the imaging criteria included in the meta-analysis varied from noncontrast computed tomography alone to computed tomography perfusion imaging or brain MRI. Previous studies did not follow the imaging criteria for “imaging mismatch,” resulting in many WUS patients not receiving timely rt-PA intravenous thrombolysis. This difference may lead to heterogeneity and bias in the final results. On the other hand, retrospective studies have limitations, such as the fact that most of the included studies did not specify the number of losses to follow-up or the rate of loss to follow-up, which may have interfered with our analysis of the results. Based on the above discussion, our clinicians should inform WUS patients and their relatives of the risk of SICH before thrombolysis.

Further limitations should be noted. First, most of the studies we included were nonrandomized because of the lack of randomized controlled trials for WUS patients both domestically and abroad. Second, the sample size of the included studies was small, and the samples were from different regions, which influenced the determination of certain indicators in the forest plots and funnel plots. which affected the determination of forest plot and funnel plot. Third, the time window of rt-PA intravenous thrombolysis in WUS patients was not uniform among the included studies. Fourth, almost all studies in this meta-analysis were lack of demographic information leading to associated bias. Finally, as the pre-examination imaging methods were not consistent across all the included studies, the pooled different measures may have led to some misrepresented results in this analysis.

## 5 Conclusion

WUS patients who received rt-PA intravenous thrombolysis had a significantly better prognosis at 90 days than those who did not receive thrombolysis, and there was no significant increase in mortality. Moreover, it is undeniable that the possibility of rt-PA intravenous thrombolysis treatment should be actively considered for WUS patients as early as possible and monitor closely hemorrhagic transformation. Finally, due to limitations of retrospective studies and the potential heterogeneity among the included studies, the results of current analysis should be further confirmed by new and larger randomized controlled trials in the future.

## Acknowledgments

The authors thank AJE for edits and revision of this manuscript.

## Author contributions

**Conceptualization:** Ying Huang.

**Data curation:** Weihua Hu, Fang Zhang, Wei Gu, Jiankun Hong.

**Methodology:** Ying Huang.

**Resources:** Ying Huang.

**Software:** Hongfa Liu.

**Writing – original draft:** Hongfa Liu.

**Writing – review & editing:** Weihua Hu, Jianping Chen, Ying Huang, Huoying Pan.
